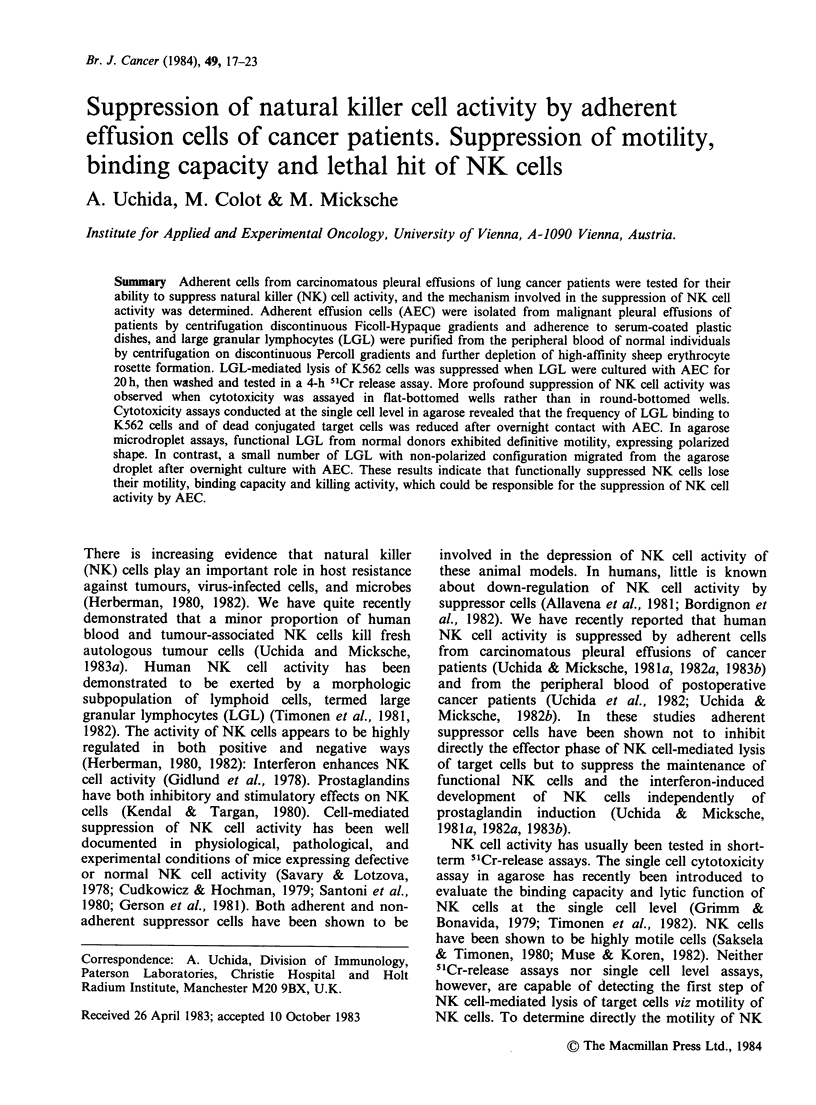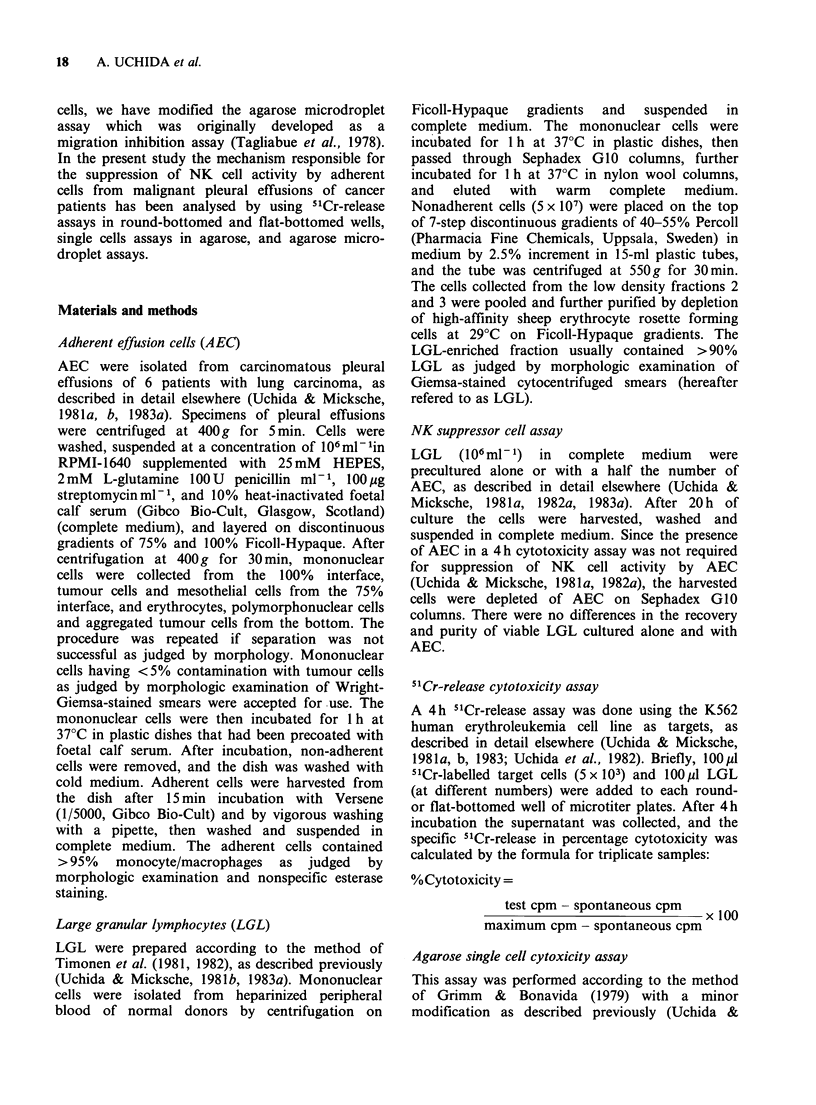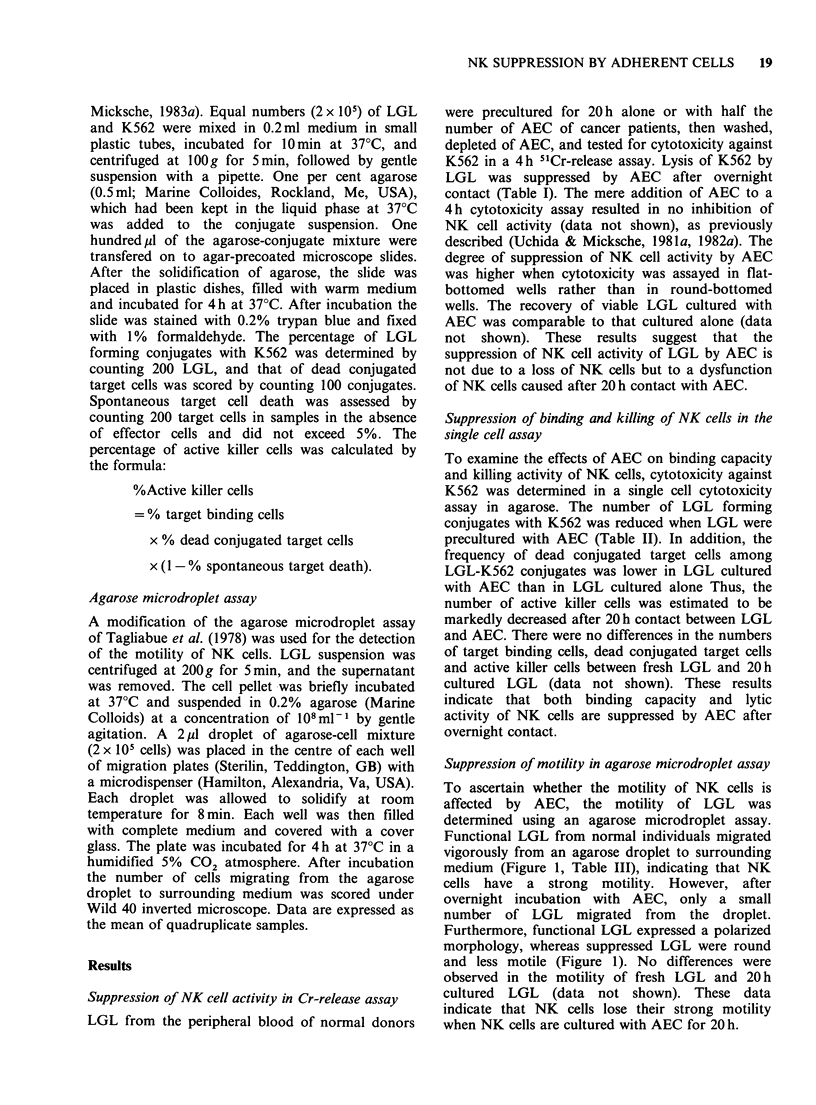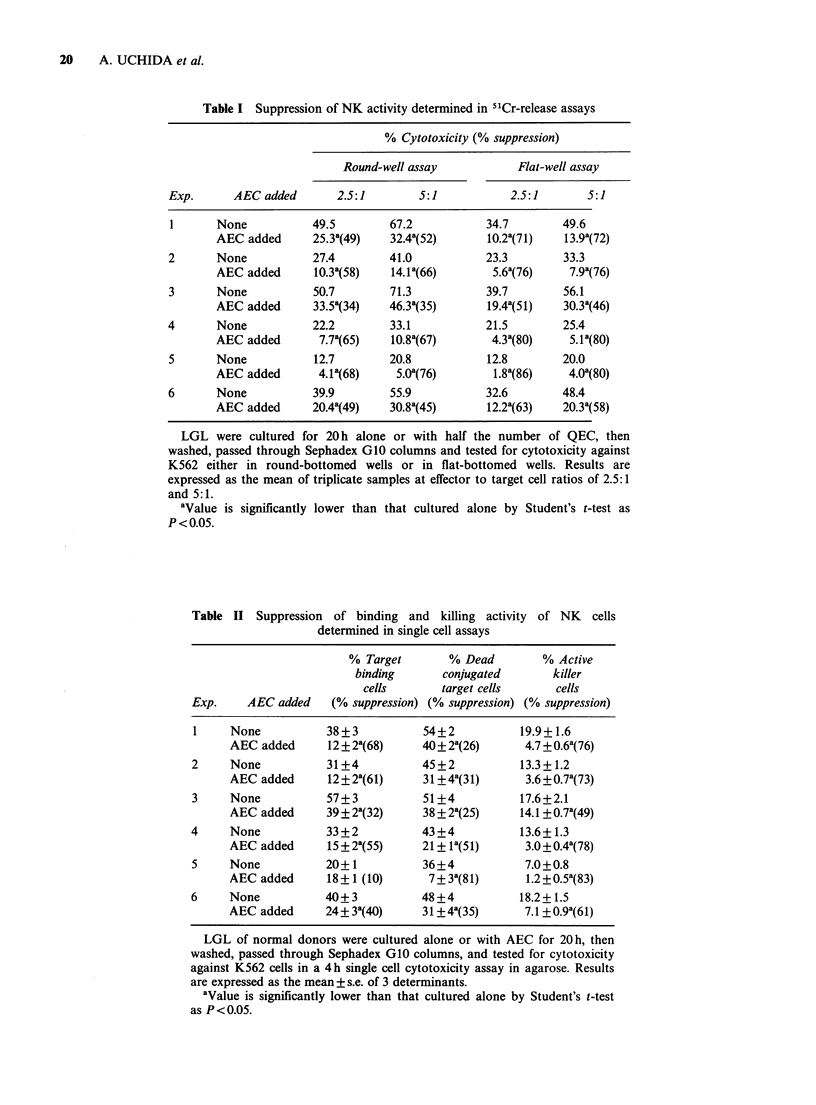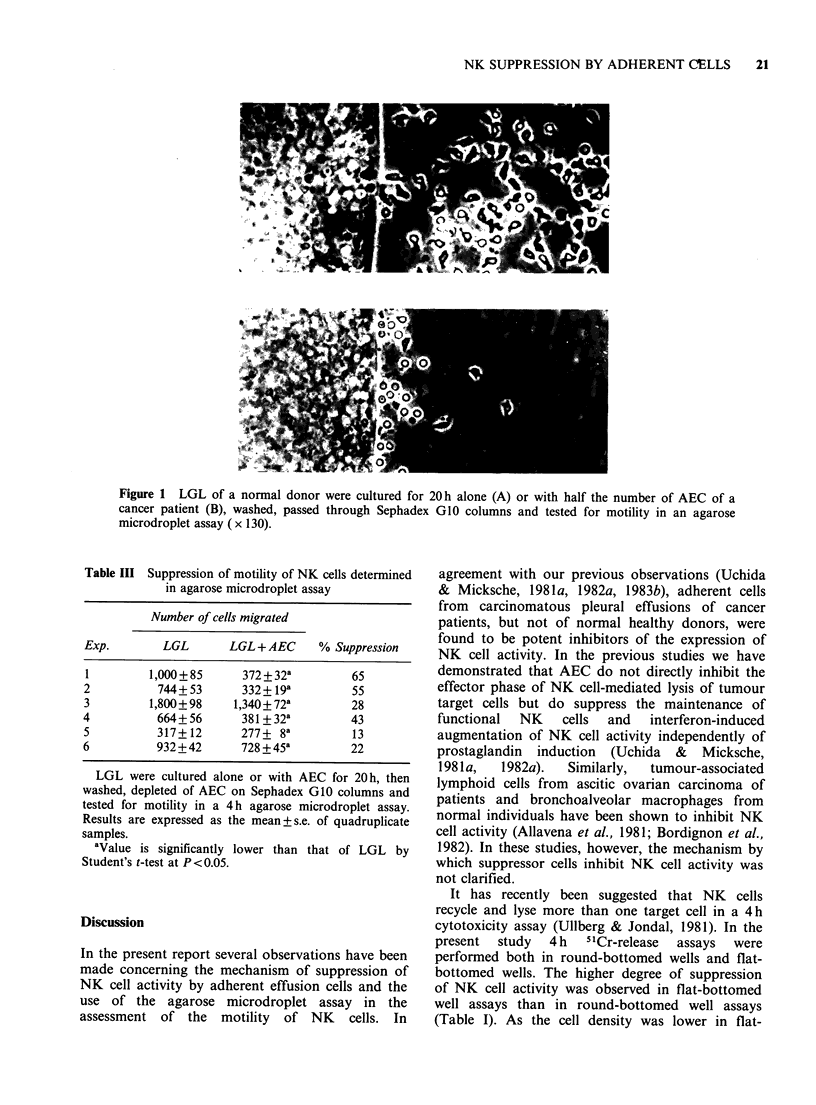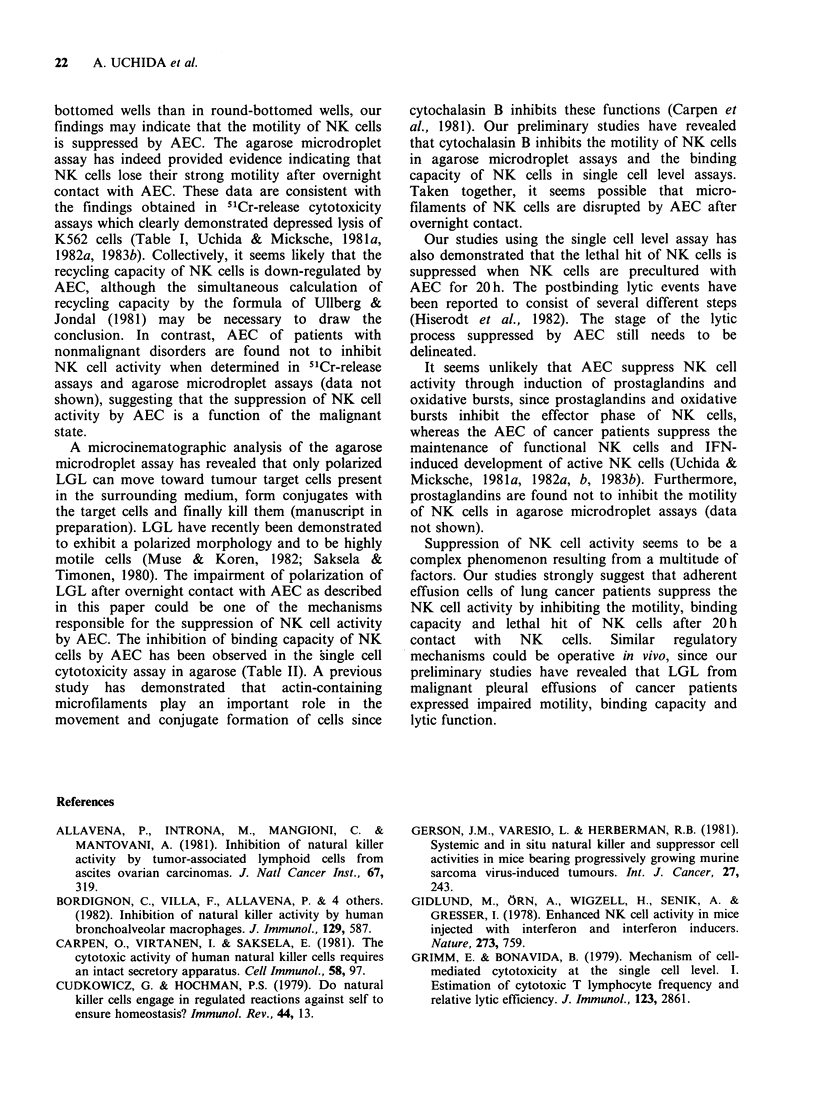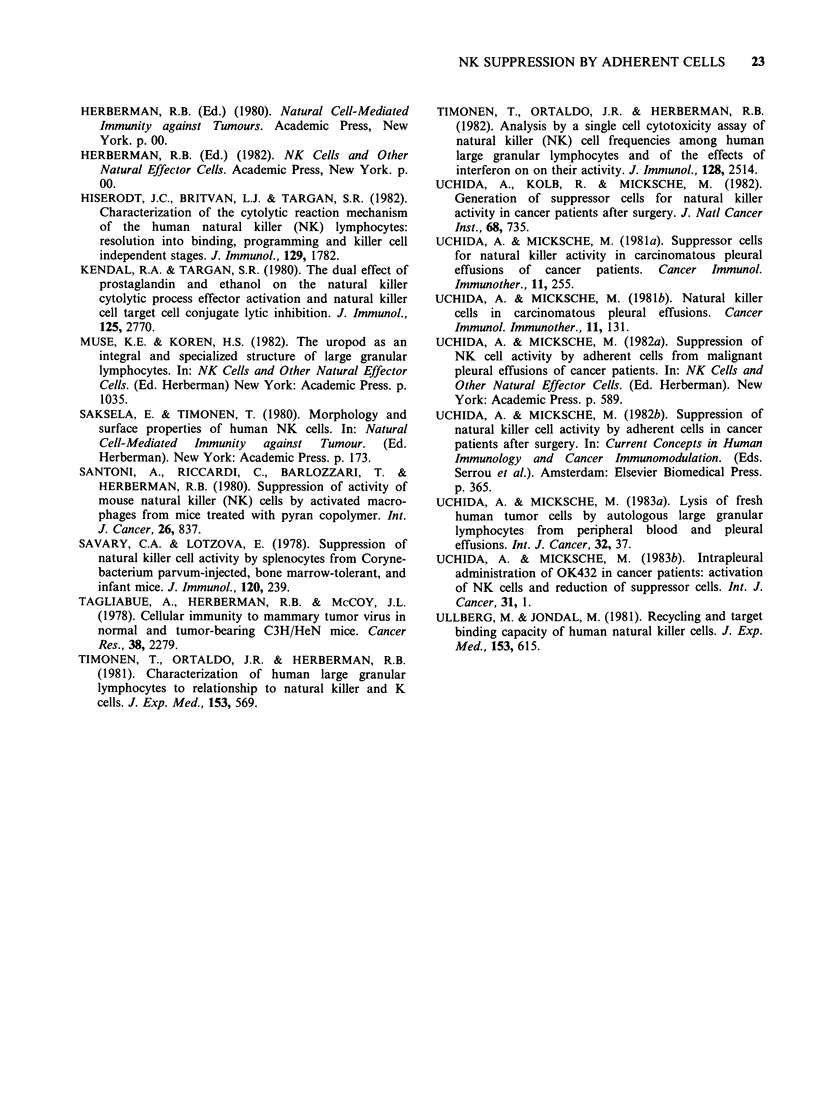# Suppression of natural killer cell activity by adherent effusion cells of cancer patients. Suppression of motility, binding capacity and lethal hit of NK cells.

**DOI:** 10.1038/bjc.1984.4

**Published:** 1984-01

**Authors:** A. Uchida, M. Colot, M. Micksche

## Abstract

**Images:**


*Br. J. Cancer* (1984), **49**, 17–23

### Suppression of natural killer cell activity by adherent effusion cells of cancer patients. Suppression of motility, binding capacity and lethal hit of NK cells

A. Uchida, M. Colot & M. Micksche

*Institute for Applied and Experimental Oncology, University of Vienna, A-1090 Vienna, Austria.*

**Summary** Adherent cells from carcinomatous pleural effusions of lung cancer patients were tested for their ability to suppress natural killer (NK) cell activity, and the mechanism involved in the suppression of NK cell activity was determined. Adherent effusion cells (AEC) were isolated from malignant pleural effusions of patients by centrifugation discontinuous Ficoll-Hypaque gradients and adherence to serum-coated plastic dishes, and large granular lymphocytes (LGL) were purified from the peripheral blood of normal individuals by centrifugation on discontinuous Percoll gradients and further depletion of high-affinity sheep erythrocyte rosette formation. LGL-mediated lysis of K562 cells was suppressed when LGL were cultured with AEC for 20 h, then washed and tested in a 4-h $^{51}Cr$ release assay. More profound suppression of NK cell activity was observed when cytotoxicity was assayed in flat-bottomed wells rather than in round-bottomed wells. Cytotoxicity assays conducted at the single cell level in agarose revealed that the frequency of LGL binding to K562 cells and of dead conjugated target cells was reduced after overnight contact with AEC. In agarose microdroplet assays, functional LGL from normal donors exhibited definitive motility, expressing polarized shape. In contrast, a small number of LGL with non-polarized configuration migrated from the agarose droplet after overnight culture with AEC. These results indicate that functionally suppressed NK cells lose their motility, binding capacity and killing activity, which could be responsible for the suppression of NK cell activity by AEC.

There is increasing evidence that natural killer (NK) cells play an important role in host resistance against tumours, virus-infected cells, and microbes (Herberman, 1980, 1982). We have quite recently demonstrated that a minor proportion of human blood and tumour-associated NK cells kill fresh autologous tumour cells (Uchida and Micksche, 1983*a*). Human NK cell activity has been demonstrated to be exerted by a morphologic subpopulation of lymphoid cells, termed large granular lymphocytes (LGL) (Timonen *et al.*, 1981, 1982). The activity of NK cells appears to be highly regulated in both positive and negative ways (Herberman, 1980, 1982): Interferon enhances NK cell activity (Gidlund *et al.*, 1978). Prostaglandins have both inhibitory and stimulatory effects on NK cells (Kendal & Targan, 1980). Cell-mediated suppression of NK cell activity has been well documented in physiological, pathological, and experimental conditions of mice expressing defective or normal NK cell activity (Savary & Lotzova, 1978; Cudkowicz & Hochman, 1979; Santoni *et al.*, 1980; Gerson *et al.*, 1981). Both adherent and non-adherent suppressor cells have been shown to be involved in the depression of NK cell activity of these animal models. In humans, little is known about down-regulation of NK cell activity by suppressor cells (Allavena *et al.*, 1981; Bordignon *et al.*, 1982). We have recently reported that human NK cell activity is suppressed by adherent cells from carcinomatous pleural effusions of cancer patients (Uchida & Micksche, 1981*a*, 1982*a*, 1983*b*) and from the peripheral blood of postoperative cancer patients (Uchida *et al.*, 1982; Uchida & Micksche, 1982*b*). In these studies adherent suppressor cells have been shown not to inhibit directly the effector phase of NK cell-mediated lysis of target cells but to suppress the maintenance of functional NK cells and the interferon-induced development of NK cells independently of prostaglandin induction (Uchida & Micksche, 1981*a*, 1982*a*, 1983*b*).

NK cell activity has usually been tested in short-term $^{51}Cr$-release assays. The single cell cytotoxicity assay in agarose has recently been introduced to evaluate the binding capacity and lytic function of NK cells at the single cell level (Grimm & Bonavida, 1979; Timonen *et al.*, 1982). NK cells have been shown to be highly motile cells (Saksela & Timonen, 1980; Muse & Koren, 1982). Neither $^{51}Cr$-release assays nor single cell level assays, however, are capable of detecting the first step of NK cell-mediated lysis of target cells *viz* motility of NK cells. To determine directly the motility of NK

Correspondence: A. Uchida, Division of Immunology, Paterson Laboratories, Christie Hospital and Holt Radium Institute, Manchester M20 9BX, U.K.

Received 26 April 1983; accepted 10 October 1983

© The Macmillan Press Ltd., 1984

cells, we have modified the agarose microdroplet assay which was originally developed as a migration inhibition assay (Tagliabue *et al.*, 1978). In the present study the mechanism responsible for the suppression of NK cell activity by adherent cells from malignant pleural effusions of cancer patients has been analysed by using $^{51}$Cr-release assays in round-bottomed and flat-bottomed wells, single cells assays in agarose, and agarose micro-droplet assays.

### Materials and methods

### *Adherent effusion cells (AEC)*

AEC were isolated from carcinomatous pleural effusions of 6 patients with lung carcinoma, as described in detail elsewhere (Uchida & Micksche, 1981*a*, *b*, 1983*a*). Specimens of pleural effusions were centrifuged at 400 *g* for 5 min. Cells were washed, suspended at a concentration of $10^6$ ml$^{-1}$in RPMI-1640 supplemented with 25 mM HEPES, 2 mM L-glutamine 100 U penicillin ml$^{-1}$, 100 μg streptomycin ml$^{-1}$, and 10% heat-inactivated foetal calf serum (Gibco Bio-Cult, Glasgow, Scotland) (complete medium), and layered on discontinuous gradients of 75% and 100% Ficoll-Hypaque. After centrifugation at 400 *g* for 30 min, mononuclear cells were collected from the 100% interface, tumour cells and mesothelial cells from the 75% interface, and erythrocytes, polymorphonuclear cells and aggregated tumour cells from the bottom. The procedure was repeated if separation was not successful as judged by morphology. Mononuclear cells having <5% contamination with tumour cells as judged by morphologic examination of Wright-Giemsa-stained smears were accepted for use. The mononuclear cells were then incubated for 1 h at 37°C in plastic dishes that had been precoated with foetal calf serum. After incubation, non-adherent cells were removed, and the dish was washed with cold medium. Adherent cells were harvested from the dish after 15 min incubation with Versene (1/5000, Gibco Bio-Cult) and by vigorous washing with a pipette, then washed and suspended in complete medium. The adherent cells contained >95% monocyte/macrophages as judged by morphologic examination and nonspecific esterase staining.

### *Large granular lymphocytes (LGL)*

LGL were prepared according to the method of Timonen *et al.* (1981, 1982), as described previously (Uchida & Micksche, 1981*b*, 1983*a*). Mononuclear cells were isolated from heparinized peripheral blood of normal donors by centrifugation on Ficoll-Hypaque gradients and suspended in complete medium. The mononuclear cells were incubated for 1 h at 37°C in plastic dishes, then passed through Sephadex G10 columns, further incubated for 1 h at 37°C in nylon wool columns, and eluted with warm complete medium. Nonadherent cells ($5 \times 10^7$) were placed on the top of 7-step discontinuous gradients of 40–55% Percoll (Pharmacia Fine Chemicals, Uppsala, Sweden) in medium by 2.5% increment in 15-ml plastic tubes, and the tube was centrifuged at 550 *g* for 30 min. The cells collected from the low density fractions 2 and 3 were pooled and further purified by depletion of high-affinity sheep erythrocyte rosette forming cells at 29°C on Ficoll-Hypaque gradients. The LGL-enriched fraction usually contained >90% LGL as judged by morphologic examination of Giemsa-stained cytocentrifuged smears (hereafter refered to as LGL).

### *NK suppressor cell assay*

LGL ($10^6$ ml$^{-1}$) in complete medium were precultured alone or with a half the number of AEC, as described in detail elsewhere (Uchida & Micksche, 1981*a*, 1982*a*, 1983*a*). After 20 h of culture the cells were harvested, washed and suspended in complete medium. Since the presence of AEC in a 4 h cytotoxicity assay was not required for suppression of NK cell activity by AEC (Uchida & Micksche, 1981*a*, 1982*a*), the harvested cells were depleted of AEC on Sephadex G10 columns. There were no differences in the recovery and purity of viable LGL cultured alone and with AEC.

### *$^{51}$Cr-release cytotoxicity assay*

A 4 h $^{51}$Cr-release assay was done using the K562 human erythroleukemia cell line as targets, as described in detail elsewhere (Uchida & Micksche, 1981*a*, b, 1983; Uchida *et al.*, 1982). Briefly, 100 μl $^{51}$Cr-labelled target cells ($5 \times 10^3$) and 100 μl LGL (at different numbers) were added to each round- or flat-bottomed well of microtiter plates. After 4 h incubation the supernatant was collected, and the specific $^{51}$Cr-release in percentage cytotoxicity was calculated by the formula for triplicate samples:

$$\%\text{Cytotoxicity} = \frac{\text{test cpm} - \text{spontaneous cpm}}{\text{maximum cpm} - \text{spontaneous cpm}} \times 100$$

### *Agarose single cell cytoxicity assay*

This assay was performed according to the method of Grimm & Bonavida (1979) with a minor modification as described previously (Uchida &

Micksche, 1983*a*). Equal numbers ($2 \times 10^5$) of LGL and K562 were mixed in 0.2 ml medium in small plastic tubes, incubated for 10 min at 37°C, and centrifuged at 100 *g* for 5 min, followed by gentle suspension with a pipette. One per cent agarose (0.5 ml; Marine Colloides, Rockland, Me, USA), which had been kept in the liquid phase at 37°C was added to the conjugate suspension. One hundred μl of the agarose-conjugate mixture were transfered on to agar-precoated microscope slides. After the solidification of agarose, the slide was placed in plastic dishes, filled with warm medium and incubated for 4 h at 37°C. After incubation the slide was stained with 0.2% trypan blue and fixed with 1% formaldehyde. The percentage of LGL forming conjugates with K562 was determined by counting 200 LGL, and that of dead conjugated target cells was scored by counting 100 conjugates. Spontaneous target cell death was assessed by counting 200 target cells in samples in the absence of effector cells and did not exceed 5%. The percentage of active killer cells was calculated by the formula:

$$\begin{aligned}\%\text{Active killer cells} &= \%\text{ target binding cells}\\ &\times \%\text{ dead conjugated target cells}\\ &\times (1 - \%\text{ spontaneous target death}).\end{aligned}$$

*Agarose microdroplet assay*

A modification of the agarose microdroplet assay of Tagliabue *et al.* (1978) was used for the detection of the motility of NK cells. LGL suspension was centrifuged at 200 *g* for 5 min, and the supernatant was removed. The cell pellet was briefly incubated at 37°C and suspended in 0.2% agarose (Marine Colloids) at a concentration of $10^8\,ml^{-1}$ by gentle agitation. A 2 μl droplet of agarose-cell mixture ($2 \times 10^5$ cells) was placed in the centre of each well of migration plates (Sterilin, Teddington, GB) with a microdispenser (Hamilton, Alexandria, Va, USA). Each droplet was allowed to solidify at room temperature for 8 min. Each well was then filled with complete medium and covered with a cover glass. The plate was incubated for 4 h at 37°C in a humidified 5% $CO_2$ atmosphere. After incubation the number of cells migrating from the agarose droplet to surrounding medium was scored under Wild 40 inverted microscope. Data are expressed as the mean of quadruplicate samples.

### Results

*Suppression of NK cell activity in Cr-release assay*

LGL from the peripheral blood of normal donors were precultured for 20 h alone or with half the number of AEC of cancer patients, then washed, depleted of AEC, and tested for cytotoxicity against K562 in a 4 h $^{51}Cr$-release assay. Lysis of K562 by LGL was suppressed by AEC after overnight contact (Table I). The mere addition of AEC to a 4 h cytotoxicity assay resulted in no inhibition of NK cell activity (data not shown), as previously described (Uchida & Micksche, 1981*a*, 1982*a*). The degree of suppression of NK cell activity by AEC was higher when cytotoxicity was assayed in flat-bottomed wells rather than in round-bottomed wells. The recovery of viable LGL cultured with AEC was comparable to that cultured alone (data not shown). These results suggest that the suppression of NK cell activity of LGL by AEC is not due to a loss of NK cells but to a dysfunction of NK cells caused after 20 h contact with AEC.

*Suppression of binding and killing of NK cells in the single cell assay*

To examine the effects of AEC on binding capacity and killing activity of NK cells, cytotoxicity against K562 was determined in a single cell cytotoxicity assay in agarose. The number of LGL forming conjugates with K562 was reduced when LGL were precultured with AEC (Table II). In addition, the frequency of dead conjugated target cells among LGL-K562 conjugates was lower in LGL cultured with AEC than in LGL cultured alone Thus, the number of active killer cells was estimated to be markedly decreased after 20 h contact between LGL and AEC. There were no differences in the numbers of target binding cells, dead conjugated target cells and active killer cells between fresh LGL and 20 h cultured LGL (data not shown). These results indicate that both binding capacity and lytic activity of NK cells are suppressed by AEC after overnight contact.

*Suppression of motility in agarose microdroplet assay*

To ascertain whether the motility of NK cells is affected by AEC, the motility of LGL was determined using an agarose microdroplet assay. Functional LGL from normal individuals migrated vigorously from an agarose droplet to surrounding medium (Figure 1, Table III), indicating that NK cells have a strong motility. However, after overnight incubation with AEC, only a small number of LGL migrated from the droplet. Furthermore, functional LGL expressed a polarized morphology, whereas suppressed LGL were round and less motile (Figure 1). No differences were observed in the motility of fresh LGL and 20 h cultured LGL (data not shown). These data indicate that NK cells lose their strong motility when NK cells are cultured with AEC for 20 h.

**Table I** Suppression of NK activity determined in $^{51}$Cr-release assays

| | | % Cytotoxicity (% suppression) | | | |
|---|---|---|---|---|---|
| | | *Round-well assay* | | *Flat-well assay* | |
| *Exp.* | *AEC added* | *2.5:1* | *5:1* | *2.5:1* | *5:1* |
| 1 | None | 49.5 | 67.2 | 34.7 | 49.6 |
| | AEC added | 25.3[a](49) | 32.4[a](52) | 10.2[a](71) | 13.9[a](72) |
| 2 | None | 27.4 | 41.0 | 23.3 | 33.3 |
| | AEC added | 10.3[a](58) | 14.1[a](66) | 5.6[a](76) | 7.9[a](76) |
| 3 | None | 50.7 | 71.3 | 39.7 | 56.1 |
| | AEC added | 33.5[a](34) | 46.3[a](35) | 19.4[a](51) | 30.3[a](46) |
| 4 | None | 22.2 | 33.1 | 21.5 | 25.4 |
| | AEC added | 7.7[a](65) | 10.8[a](67) | 4.3[a](80) | 5.1[a](80) |
| 5 | None | 12.7 | 20.8 | 12.8 | 20.0 |
| | AEC added | 4.1[a](68) | 5.0[a](76) | 1.8[a](86) | 4.0[a](80) |
| 6 | None | 39.9 | 55.9 | 32.6 | 48.4 |
| | AEC added | 20.4[a](49) | 30.8[a](45) | 12.2[a](63) | 20.3[a](58) |

LGL were cultured for 20 h alone or with half the number of QEC, then washed, passed through Sephadex G10 columns and tested for cytotoxicity against K562 either in round-bottomed wells or in flat-bottomed wells. Results are expressed as the mean of triplicate samples at effector to target cell ratios of 2.5:1 and 5:1.

[a]Value is significantly lower than that cultured alone by Student's *t*-test as $P<0.05$.

**Table II** Suppression of binding and killing activity of NK cells determined in single cell assays

| *Exp.* | *AEC added* | % *Target binding cells* (% *suppression*) | % *Dead conjugated target cells* (% *suppression*) | % *Active killer cells* (% *suppression*) |
|---|---|---|---|---|
| 1 | None | 38±3 | 54±2 | 19.9±1.6 |
| | AEC added | 12±2[a](68) | 40±2[a](26) | 4.7±0.6[a](76) |
| 2 | None | 31±4 | 45±2 | 13.3±1.2 |
| | AEC added | 12±2[a](61) | 31±4[a](31) | 3.6±0.7[a](73) |
| 3 | None | 57±3 | 51±4 | 17.6±2.1 |
| | AEC added | 39±2[a](32) | 38±2[a](25) | 14.1±0.7[a](49) |
| 4 | None | 33±2 | 43±4 | 13.6±1.3 |
| | AEC added | 15±2[a](55) | 21±1[a](51) | 3.0±0.4[a](78) |
| 5 | None | 20±1 | 36±4 | 7.0±0.8 |
| | AEC added | 18±1 (10) | 7±3[a](81) | 1.2±0.5[a](83) |
| 6 | None | 40±3 | 48±4 | 18.2±1.5 |
| | AEC added | 24±3[a](40) | 31±4[a](35) | 7.1±0.9[a](61) |

LGL of normal donors were cultured alone or with AEC for 20 h, then washed, passed through Sephadex G10 columns, and tested for cytotoxicity against K562 cells in a 4 h single cell cytotoxicity assay in agarose. Results are expressed as the mean±s.e. of 3 determinants.

[a]Value is significantly lower than that cultured alone by Student's *t*-test as $P<0.05$.

**Figure 1** LGL of a normal donor were cultured for 20 h alone (A) or with half the number of AEC of a cancer patient (B), washed, passed through Sephadex G10 columns and tested for motility in an agarose microdroplet assay ( × 130).

**Table III** Suppression of motility of NK cells determined in agarose microdroplet assay

| | *Number of cells migrated* | | |
|---|---|---|---|
| *Exp.* | *LGL* | *LGL+AEC* | *% Suppression* |
| 1 | 1,000±85 | 372±32[a] | 65 |
| 2 | 744±53 | 332±19[a] | 55 |
| 3 | 1,800±98 | 1,340±72[a] | 28 |
| 4 | 664±56 | 381±32[a] | 43 |
| 5 | 317±12 | 277± 8[a] | 13 |
| 6 | 932±42 | 728±45[a] | 22 |

LGL were cultured alone or with AEC for 20 h, then washed, depleted of AEC on Sephadex G10 columns and tested for motility in a 4 h agarose microdroplet assay. Results are expressed as the mean±s.e. of quadruplicate samples.

[a]Value is significantly lower than that of LGL by Student's *t*-test at $P<0.05$.

### Discussion

In the present report several observations have been made concerning the mechanism of suppression of NK cell activity by adherent effusion cells and the use of the agarose microdroplet assay in the assessment of the motility of NK cells. In agreement with our previous observations (Uchida & Micksche, 1981*a*, 1982*a*, 1983*b*), adherent cells from carcinomatous pleural effusions of cancer patients, but not of normal healthy donors, were found to be potent inhibitors of the expression of NK cell activity. In the previous studies we have demonstrated that AEC do not directly inhibit the effector phase of NK cell-mediated lysis of tumour target cells but do suppress the maintenance of functional NK cells and interferon-induced augmentation of NK cell activity independently of prostaglandin induction (Uchida & Micksche, 1981*a*, 1982*a*). Similarly, tumour-associated lymphoid cells from ascitic ovarian carcinoma of patients and bronchoalveolar macrophages from normal individuals have been shown to inhibit NK cell activity (Allavena *et al.*, 1981; Bordignon *et al.*, 1982). In these studies, however, the mechanism by which suppressor cells inhibit NK cell activity was not clarified.

It has recently been suggested that NK cells recycle and lyse more than one target cell in a 4 h cytotoxicity assay (Ullberg & Jondal, 1981). In the present study 4 h $^{51}Cr$-release assays were performed both in round-bottomed wells and flat-bottomed wells. The higher degree of suppression of NK cell activity was observed in flat-bottomed well assays than in round-bottomed well assays (Table I). As the cell density was lower in flatbottomed wells than in round-bottomed wells, our findings may indicate that the motility of NK cells is suppressed by AEC. The agarose microdroplet assay has indeed provided evidence indicating that NK cells lose their strong motility after overnight contact with AEC. These data are consistent with the findings obtained in $^{51}$Cr-release cytotoxicity assays which clearly demonstrated depressed lysis of K562 cells (Table I, Uchida & Micksche, 1981*a*, 1982*a*, 1983*b*). Collectively, it seems likely that the recycling capacity of NK cells is down-regulated by AEC, although the simultaneous calculation of recycling capacity by the formula of Ullberg & Jondal (1981) may be necessary to draw the conclusion. In contrast, AEC of patients with nonmalignant disorders are found not to inhibit NK cell activity when determined in $^{51}$Cr-release assays and agarose microdroplet assays (data not shown), suggesting that the suppression of NK cell activity by AEC is a function of the malignant state.

A microcinematographic analysis of the agarose microdroplet assay has revealed that only polarized LGL can move toward tumour target cells present in the surrounding medium, form conjugates with the target cells and finally kill them (manuscript in preparation). LGL have recently been demonstrated to exhibit a polarized morphology and to be highly motile cells (Muse & Koren, 1982; Saksela & Timonen, 1980). The impairment of polarization of LGL after overnight contact with AEC as described in this paper could be one of the mechanisms responsible for the suppression of NK cell activity by AEC. The inhibition of binding capacity of NK cells by AEC has been observed in the single cell cytotoxicity assay in agarose (Table II). A previous study has demonstrated that actin-containing microfilaments play an important role in the movement and conjugate formation of cells since cytochalasin B inhibits these functions (Carpen *et al.*, 1981). Our preliminary studies have revealed that cytochalasin B inhibits the motility of NK cells in agarose microdroplet assays and the binding capacity of NK cells in single cell level assays. Taken together, it seems possible that microfilaments of NK cells are disrupted by AEC after overnight contact.

Our studies using the single cell level assay has also demonstrated that the lethal hit of NK cells is suppressed when NK cells are precultured with AEC for 20 h. The postbinding lytic events have been reported to consist of several different steps (Hiserodt *et al.*, 1982). The stage of the lytic process suppressed by AEC still needs to be delineated.

It seems unlikely that AEC suppress NK cell activity through induction of prostaglandins and oxidative bursts, since prostaglandins and oxidative bursts inhibit the effector phase of NK cells, whereas the AEC of cancer patients suppress the maintenance of functional NK cells and IFN-induced development of active NK cells (Uchida & Micksche, 1981*a*, 1982*a*, *b*, 1983*b*). Furthermore, prostaglandins are found not to inhibit the motility of NK cells in agarose microdroplet assays (data not shown).

Suppression of NK cell activity seems to be a complex phenomenon resulting from a multitude of factors. Our studies strongly suggest that adherent effusion cells of lung cancer patients suppress the NK cell activity by inhibiting the motility, binding capacity and lethal hit of NK cells after 20 h contact with NK cells. Similar regulatory mechanisms could be operative *in vivo*, since our preliminary studies have revealed that LGL from malignant pleural effusions of cancer patients expressed impaired motility, binding capacity and lytic function.

### References

ALLAVENA, P., INTRONA, M., MANGIONI, C. & MANTOVANI, A. (1981). Inhibition of natural killer activity by tumor-associated lymphoid cells from ascites ovarian carcinomas. *J. Natl Cancer Inst.*, **67**, 319.

BORDIGNON, C., VILLA, F., ALLAVENA, P. & 4 others. (1982). Inhibition of natural killer activity by human bronchoalveolar macrophages. *J. Immunol.*, **129**, 587.

CARPEN, O., VIRTANEN, I. & SAKSELA, E. (1981). The cytotoxic activity of human natural killer cells requires an intact secretory apparatus. *Cell Immunol.*, **58**, 97.

CUDKOWICZ, G. & HOCHMAN, P.S. (1979). Do natural killer cells engage in regulated reactions against self to ensure homeostasis? *Immunol. Rev.*, **44**, 13.

GERSON, J.M., VARESIO, L. & HERBERMAN, R.B. (1981). Systemic and in situ natural killer and suppressor cell activities in mice bearing progressively growing murine sarcoma virus-induced tumours. *Int. J. Cancer*, **27**, 243.

GIDLUND, M., ÖRN, A., WIGZELL, H., SENIK, A. & GRESSER, I. (1978). Enhanced NK cell activity in mice injected with interferon and interferon inducers. *Nature*, **273**, 759.

GRIMM, E. & BONAVIDA, B. (1979). Mechanism of cell-mediated cytotoxicity at the single cell level. I. Estimation of cytotoxic T lymphocyte frequency and relative lytic efficiency. *J. Immunol.*, **123**, 2861.

HERBERMAN, R.B. (Ed.) (1980). *Natural Cell-Mediated Immunity against Tumours*. Academic Press, New York. p. 00.

HERBERMAN, R.B. (Ed.) (1982). *NK Cells and Other Natural Effector Cells*. Academic Press, New York. p. 00.

HISERODT, J.C., BRITVAN, L.J. & TARGAN, S.R. (1982). Characterization of the cytolytic reaction mechanism of the human natural killer (NK) lymphocytes: resolution into binding, programming and killer cell independent stages. *J. Immunol.*, **129**, 1782.

KENDAL, R.A. & TARGAN, S.R. (1980). The dual effect of prostaglandin and ethanol on the natural killer cytolytic process effector activation and natural killer cell target cell conjugate lytic inhibition. *J. Immunol.*, **125**, 2770.

MUSE, K.E. & KOREN, H.S. (1982). The uropod as an integral and specialized structure of large granular lymphocytes. In: *NK Cells and Other Natural Effector Cells*. (Ed. Herberman) New York: Academic Press. p. 1035.

SAKSELA, E. & TIMONEN, T. (1980). Morphology and surface properties of human NK cells. In: *Natural Cell-Mediated Immunity against Tumour*. (Ed. Herberman). New York: Academic Press. p. 173.

SANTONI, A., RICCARDI, C., BARLOZZARI, T. & HERBERMAN, R.B. (1980). Suppression of activity of mouse natural killer (NK) cells by activated macrophages from mice treated with pyran copolymer. *Int. J. Cancer*, **26**, 837.

SAVARY, C.A. & LOTZOVA, E. (1978). Suppression of natural killer cell activity by splenocytes from Corynebacterium parvum-injected, bone marrow-tolerant, and infant mice. *J. Immunol.*, **120**, 239.

TAGLIABUE, A., HERBERMAN, R.B. & McCOY, J.L. (1978). Cellular immunity to mammary tumor virus in normal and tumor-bearing C3H/HeN mice. *Cancer Res.*, **38**, 2279.

TIMONEN, T., ORTALDO, J.R. & HERBERMAN, R.B. (1981). Characterization of human large granular lymphocytes to relationship to natural killer and K cells. *J. Exp. Med.*, **153**, 569.

TIMONEN, T., ORTALDO, J.R. & HERBERMAN, R.B. (1982). Analysis by a single cell cytotoxicity assay of natural killer (NK) cell frequencies among human large granular lymphocytes and of the effects of interferon on on their activity. *J. Immunol.*, **128**, 2514.

UCHIDA, A., KOLB, R. & MICKSCHE, M. (1982). Generation of suppressor cells for natural killer activity in cancer patients after surgery. *J. Natl Cancer Inst.*, **68**, 735.

UCHIDA, A. & MICKSCHE, M. (1981*a*). Suppressor cells for natural killer activity in carcinomatous pleural effusions of cancer patients. *Cancer Immunol. Immunother.*, **11**, 255.

UCHIDA, A. & MICKSCHE, M. (1981*b*). Natural killer cells in carcinomatous pleural effusions. *Cancer Immunol. Immunother.*, **11**, 131.

UCHIDA, A. & MICKSCHE, M. (1982*a*). Suppression of NK cell activity by adherent cells from malignant pleural effusions of cancer patients. In: *NK Cells and Other Natural Effector Cells*. (Ed. Herberman). New York: Academic Press. p. 589.

UCHIDA, A. & MICKSCHE, M. (1982*b*). Suppression of natural killer cell activity by adherent cells in cancer patients after surgery. In: *Current Concepts in Human Immunology and Cancer Immunomodulation*. (Eds. Serrou *et al.*). Amsterdam: Elsevier Biomedical Press. p. 365.

UCHIDA, A. & MICKSCHE, M. (1983*a*). Lysis of fresh human tumor cells by autologous large granular lymphocytes from peripheral blood and pleural effusions. *Int. J. Cancer*, **32**, 37.

UCHIDA, A. & MICKSCHE, M. (1983*b*). Intrapleural administration of OK432 in cancer patients: activation of NK cells and reduction of suppressor cells. *Int. J. Cancer*, **31**, 1.

ULLBERG, M. & JONDAL, M. (1981). Recycling and target binding capacity of human natural killer cells. *J. Exp. Med.*, **153**, 615.